# Tuning the Porosity of Piezoelectric Zinc Oxide Thin Films Obtained from Molecular Layer-Deposited “Zincones”

**DOI:** 10.3390/ma15196786

**Published:** 2022-09-30

**Authors:** Marianne Kräuter, Taher Abu Ali, Barbara Stadlober, Roland Resel, Katrin Unger, Anna Maria Coclite

**Affiliations:** 1 Institute of Solid State Physics, NAWI Graz, Graz University of Technology, Petersgasse 16, 8010 Graz, Austria; 2 MATERIALS-Institute for Surface Technologies and Photonics, Joanneum Research Forschungsgesellschaft mbH, Franz-Pichler-Str. 30, 8160 Weiz, Austria

**Keywords:** ZnO, annealing, porosimetric ellipsometry, piezoresponse, porous thin film, molecular layer deposition

## Abstract

Porous zinc oxide (ZnO) thin films were synthesized via the calcination of molecular layer-deposited (MLD) “zincone” layers. The effect of the MLD process temperature (110 °C, 125 °C) and of the calcination temperature (340 °C, 400 °C, 500 °C) on the chemical, morphological, and crystallographic properties of the resulting ZnO was thoroughly investigated. Spectroscopic ellipsometry reveals that the thickness of the calcinated layers depends on the MLD temperature, resulting in 38–43% and 52–56% of remaining thickness for the 110 °C and 125 °C samples, respectively. Ellipsometric porosimetry shows that the open porosity of the ZnO thin films depends on the calcination temperature as well as on the MLD process temperature. The maximum open porosity of ZnO derived from zincone deposited at 110 °C ranges from 14.5% to 24%, rising with increasing calcination temperature. Compared with the 110 °C samples, the ZnO obtained from 125 °C zincone yields a higher porosity for low calcination temperatures, namely 18% for calcination at 340 °C; and up to 24% for calcination at 500 °C. Additionally, the porous ZnO thin films were subjected to piezoelectric measurements. The piezoelectric coefficient, d_33_, was determined to be 2.8 pC/N, demonstrating the potential of the porous ZnO as an, e.g., piezoelectric sensor or energy harvester.

## 1. Introduction

Due to the interesting optical, electrical, and piezoelectric properties of zinc oxide [1] (ZnO), thin films of this material have been studied for a variety of applications such as piezoelectric generators [2,3], transparent conductive oxides [4], or resistance random access memories [5]. Introducing porosity to ZnO thin films opens up a plethora of new possibilities by virtue of the increased surface area. Porous ZnO thin films are often employed as a host material for biomedical applications, such as tissue engineering [6] and drug delivery [7], as photocatalysts [7,8], or for gas sensing [7,9,10] as well as for various devices which make use of the piezoelectric effect (e.g., nanogenerators and force sensors [11]). A few studies have shown that porous ZnO can yield enhanced piezoelectricity compared to its nonporous counterpart [11,12].

To enable the use of porous ZnO thin films in commercial applications, a deposition technique is needed, which promises conformal growth, high controllability over film formation, and which can be scaled up easily. These requirements are met, e.g., by chemical vapor deposition techniques, which allow for the synthesis of thin films with excellent conformality [13]. In particular, molecular layer deposition (MLD) is a first-rate choice for deposition on complex high aspect ratio nanostructured substrates and devices due to its self-limiting layer-by-layer nature and provides precise thickness control in the Angström range [14].

MLD is typically employed for the delivery of (extremely) thin organic and hybrid organic–inorganic films. The ideal MLD precursors must be volatile as well as thermally stable. In order to ensure a reasonable deposition rate, they have to chemisorb on the surface or react with the present surface groups rapidly [15]. The organic precursors ideally possess a stiff aromatic backbone and small ligands to avoid tilting and to prevent steric hindrance effects, respectively. Furthermore, heterobifunctional precursors are desirable as homobifunctional precursors are prone to bend and react more than once with the available surface groups, thus reducing the number of active surface groups and consequently the overall growth rate [16]. Often, real precursors do not possess all mentioned ideal properties resulting in a reduced growth rate compared to the theoretical optimum.

A specific type of hybrid materials can be deposited by combining metalorganic precursors with the ligands utilized in organic MLD. The resulting hybrid films are generally composed of an organic backbone combined with oxygen and/or nitrogen and a metallic element [14]. These films are termed “metal-alkoxides” or “metalcones”, depending on the metallic precursor employed in their synthesis (e.g., alucone, titanicone, zircone, zincone) [17]. Several hybrid films have been reported in literature, using different metallic precursors (e.g., Al [18], Fe [19], Hf [20], Li [21], Sn [22], Ti [23,24], V [25], Zn [26,27], Zr [17]). Regarding zincones, their formation in a two-step MLD deposition process is well known, especially for the precursors diethylzinc and ethylene glycol [28,29].

Porous metal oxide thin films can be obtained from the MLD metalcones through a post-treatment step such as water etching or calcination in an oxygen atmosphere [30]. Several MLD-derived oxides have been studied in the literature, including but not limited to Al-based [31], Ti-based [32], and Mg-based [33] materials. These studies present process windows for the formation of highly porous materials.

In contrast, thorough studies regarding the synthesis of porous ZnO via calcination for vapor-deposited zincones are scarce. Liang et al. [34] studied ZnO derived from zincone layers on titania nanoparticles. The synthesis of low surface area ZnO as well as wide distribution of micro- and mesopores was reported. However, the pore content was not communicated. Moreover, the study was limited to ultrathin zincone films with a thickness of about 1 nm, as assessed via transmission electron microscopy. Previous work from our group [35] showed that zincone-like thin films can be deposited via sub-saturated plasma-enhanced atomic layer deposition and that porous ZnO can be obtained from them via calcination, albeit the porosity achieved was quite low (1–5%). More promising porosity values were reached in the following publication [36], in which the zincone thin films were deposited via MLD using diethyl zinc (DEZ) and ethylene glycol (EG) as the metal and organic precursor, respectively. Subsequent calcination at 400 °C led to an open porosity of approximately 19% in the obtained ZnO thin film. A further increase in porosity was obtained by changing to a three-step MLD process, employing DEZ as the metal precursor together with two organic precursors (maleic anhydride and ethanolamine) [37]. The calcinated samples showed an open porosity of 25% when calcinated at 400 °C.

Even though a few porosity studies regarding MLD-derived porous ZnO exist, so far several crucial factors have not been explored sufficiently or at all: The calcination temperatures explored so far are limited to 400 °C and 600 °C, and possible effects of MLD deposition parameters—such as the deposition temperature—onto the porosity have not yet been investigated at all. Furthermore, proof of concept that the porous ZnO synthesized in this fashion can indeed be used in the desired fields of application, e.g., for piezoelectric devices—has not been provided in the cited studies. Although piezoelectric characterization has been reported for ZnO films grown by different techniques [11,38,39], to the best of the authors’ knowledge, no literature on piezoelectric properties of porous ZnO thin films (thickness <1 μm) exists.

This contribution serves to expand the knowledge regarding MLD-derived porous ZnO. Additional calcination temperatures of 340 °C and 500 °C were explored to gain a more complete picture of the tunability of the porosity of the ZnO thin films. Additionally, the effect of the MLD deposition temperature (110 °C and 125 °C) was explored as an influencing factor of the ZnO thin film porosity. The porosity was investigated via ellipsometric porosimetry (EP), a technique that has already shown its usefulness for the determination of porosity in hybrid- and polymer-derived oxides [33,35,40,41,42]. Adding to the EP measurements, the porous ZnO thin films were thoroughly investigated with respect to their topographical, chemical, and crystallographic properties. Moreover, the macroscopic piezoelectric properties of the synthesized porous ZnO were assessed to showcase their potential for piezoelectric devices.

## 2. Materials and Methods

### 2.1. Synthesis of Molecular Layer-Deposited Zincone

Zincone thin films were deposited on single-side polished c-Si (100) substrates (Siegert Wafer) in a custom-built molecular layer deposition (MLD) reactor, connected to a rotary vane pump (Pfeiffer Vacuum Duo 20M). A schematic of the set-up can be found in Figure S1a. Diethyl zinc (DEZ, Dock/Chemicals, CAS-No.: 557-20-0) was employed as the metal-organic precursor, ethylene glycol (EG, Sigma-Aldrich, CAS-No.: 107-21-1) as the organic co-precursor. Both precursors were pulsed into the reactor via automated ALD-valves (Swagelok ALD3). To avoid condensation of the precursor vapors inside the system, the MLD set-up was heated as detailed in Table S1. A constant Ar-flow of 16 sccm served as the purging and carrier gas throughout the whole deposition process. A mass flow controller (MKS MF1-C) and an automation platform (MKS PAC 1000) were used to control the flow rate of the Ar. Precise control of the ALD valves was realized with in-house programmed software [43].

The MLD process included the following four steps, repeated for each cycle: (1) DEZ dose, (2) Ar purging, (3) EG dose, and (4) Ar purging. To ensure the saturation behavior of the four steps as previously investigated [36], the deposition recipe was fixed to 0.15 s for the DEZ dose, 60 s for the first Ar purge, 0.2 s for the EG exposure, and 170 s for the second Ar purge. The 16 sccm set for the Ar flow resulted in working pressure of 0.15 torr, and relative precursor pressures of Δp = (0.2 ± 0.02) torr for DEZ and of Δp = (0.1 ± 0.02) torr for EG. Exemplary pressure curves for depositions with substrate temperatures of (110 ± 2) °C and (125 ± 2) °C are shown in Figure S1b,c.

### 2.2. Synthesis of Porous ZnO from MLD Zincone

To remove the organic ligands from the zincone samples—in order to obtain porous ZnO—they were calcinated ex situ in air to 340 °C, 400 °C, and 500 °C with a heating rate of 3 °C/min using a programmable heating stage, which consisted of the heating plate (PZ20-3TD, Harry Gestigkeit GmbH, Düsseldorf, Germany) and a programmable controller (PR 53T, Harry Gestigkeit GmbH).

**Figure 1:**
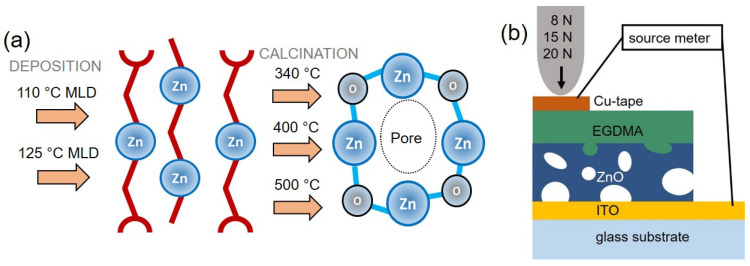
(**a**) Schematic of the processing steps used to obtain porous ZnO and (**b**) schematic of the porous ZnO samples as prepared for piezoelectric measurements. The image is not in scale.

Additionally, in situ annealing to 400 °C was performed with a spectroscopic ellipsometry system, described below in Section 2.3. The processes of deposition and calcination are represented in Figure 1a.

### 2.3. Optical Characterization

Thickness and refractive index of the zincone and ZnO thin films were determined via spectroscopic ellipsometry (SE, J.A. Woollam M-2000V, Lincoln, NE, USA). The measured data were evaluated with the software CompleteEASE (Version 5.19, 2018, J.A. Woollam) by employing a three-layer model, composed of the Si substrate, a SiO_2_ native oxide layer with a fixed thickness of 1.5 nm, and a Cauchy model representing the actual thin film.

The Cauchy model describes the real part of the refractive index n as a function of the wavelength λ and the fitting parameters. The obtained data were fitted at three different angles (65°, 70°, and 75°) in a spectral range between 400–1000 nm excluding wavelengths in the range of the ZnO bandgap (3.3 eV ≙ 376 nm). Thus, absorption effects are negligible when fitting both metal–organic hybrid and crystalline ZnO films and allow the use of the Cauchy model for transparent materials.

The growth per cycle (GPC) was discerned by averaging the thickness of at least 5 samples measured via SE and dividing this average by the number of cycles.

To follow the calcination process in situ, the SE system was equipped with a THMS600 temperature stage (Linkam, Redhill, UK), with a sealing capping chamber. The temperature increased from room temperature to 400 °C at a rate of 180 °C per hour (≙ 3 °C/min), while continuously measuring the film thickness as well as the refractive index as a function of temperature. Subsequently, the system was cooled down to room temperature again. For these experiments, the acquisition angle was fixed at 70°.

For the determination of the porosity in the porous ZnO thin films, ellipsometric porosimetry measurements were performed. The principle of this method is to measure the refractive index of a sample via SE, while simultaneously increasing the partial pressure of a probing vapor in the atmosphere surrounding the sample [44]. The ellipsometer system was modified by attaching a custom bubbler system to the THMS600 temperature stage. A schematic of the set-up can be found in Figure S2. An N_2_-flow was used to remove water from the measurement chamber and to provide a dry atmosphere. To tune the relative humidity inside the chamber, the flow of N_2_ was mixed with humid air and adjusted in equilibrium steps. The temperature of the stage was kept at room temperature throughout the experiment. The relative humidity was monitored via a sensor (Sparkfun SHT-15, Niwot, CO, USA) in the measurements chamber and tuned in the range of 0–89%. In this way, pores with a diameter ≥0.33 nm (i.e., the kinetic diameter of the water molecules) were probed. More on the theoretical background of this procedure is available in the literature [35,37,44].

### 2.4. Chemical, Crystallographic, and Morphological Characterization

Fourier transform infrared spectroscopy (FT-IR) measurements were conducted in transmission mode with a Michelson interferometer (Bruker IFS 66V, Billerica, MA, USA), in the range of 750–4000 cm^−1^, and processed in the OPUS software. The measurement environment was kept under vacuum to minimize light scattering. Reference measurements of a single side polished c–Si (100) substrates (Siegert Wafer, Aachen, Germany) were performed for background subtraction.

The crystalline properties of the films were investigated via 2θ-scans, with a Panalytical Empyrean system working in θ/θ-configuration and equipped with a copper tube (λ = 1.5418 Å). For these studies, the incidence angle was fixed at 0.2° while the detector angle was varied from 30° to 40°. A 1/32° divergence slit, and a 10 mm mask were used on the primary side of the instrument, together with a multilayer mirror and an automatic beam attenuator. Although the secondary side was equipped with a 7.5 mm anti-scatter slit and a Soller slit of 0.02 rad. The integration time per measurement was set to 1000 s with a step size of 0.0131°. The detector was operated in the 1D mode.

For additional characterization of the in-plane order of the ZnO films, grazing incidence X-ray diffraction (GIXD) measurements were performed on a commercial four-circle Bruker D8 Discover diffractometer, which had been upgraded with the Bruker Ultra GID addition. A conventional 2.2 kW water-cooled X-ray tube with a copper anode in line-focus mode was used as X-ray source. The divergent X-ray beam emitted from the line-shaped source was collimated out-of-plane by a parabolic graded multilayer mirror (Schuster & Gobel, Munich, Germany), whereas the in-plane incoming beam divergence was adjusted by Soller slits. A one-dimensional positron-sensitive detector (PSD; Vantec-1, Porto, Portugal) was used to collect scattering intensity profiles along the out-of-plane direction. More information about this experimental set-up can be found in the literature [45]. The experimental results of the GIXD measurements were visualized and analyzed using the custom-made software PyGid.

X-ray photoelectron spectroscopy (XPS) was performed in an ultrahigh vacuum (UHV) chamber equipped with a dual anode X-ray source (SPECS XR 50, Berlin, Germany) and a hemispherical energy analyzer (SPECS Phoibos 150). The samples were fixed on a Ti sample plate and transferred into the UHV chamber via a load-lock. Spectra were acquired with non-monochromatized Al Kα radiation (400 W) and evaluated with the demo version of CasaXPS (Version 2.3.24). To remove surface contaminations, the samples were sputtered for 10 min with Ar before measurement to remove surface contaminations.

Atomic force microscopy (AFM) was carried out in non-contact mode on a Nanosurf Easyscan 2, equipped with a PPP-NCLR-10 cantilever (NanoWorld AG, Neuchâtel, Switzerland). Data evaluation was performed with the freely available software package Gwyddion (Version 2.58).

### 2.5. Piezoelectric Measurements of Porous ZnO

For the piezoelectric measurements, first, zincone thin films were deposited on glass covered with 15–30 nm indium tin oxide (ITO, 8–12 Ω/cm^2^, Sigma Aldrich, CAS-No.: 50926-11-9) via a 1000 cycle MLD deposition at a process temperature of 110 °C. A small part of the ITO glass substrate was covered with Kapton tape during the deposition to keep the zincone from being deposited on the whole ITO surface and keep a portion uncoated to serve as the bottom electrode. Afterward, the samples were annealed ex situ to 400 °C as described above.

A total of 60 nm of ethylene glycol dimethacrylate (EGDMA, Sigma Aldrich, CAS-No.: 97-90-5) were deposited on top of the porous ZnO as a dielectric layer to minimize leakage current between the bottom and top electrode, via initiated chemical vapor deposition (iCVD), again covering part of the bottom electrode with Kapton. The iCVD deposition was performed in an in-house built system [46], as detailed in the Supplementary Materials. For the deposition, the filament was heated to about 250 °C while the substrate was kept at 30 °C; the jar of EGDMA itself was heated to 85 °C, and the initiator tert-butyl peroxide (TBPO, Sigma Aldrich, CAS-No.: 110-05-4) was kept at room temperature. The working pressure was set to 200 mtorr, flow rates were set to 0.15 sccm for EGDMA and to 0.8 sccm for TBPO at a working pressure of 200 mtorr. A schematic of the samples as prepared for the piezoelectric measurements can be found in Figure 1b.

The lowest leakage current between the bottom and top electrodes was obtained when the conductive copper tape was chosen as a top electrode and applied to the ZnO-pEGDMA film. The macroscopic piezoelectric properties were measured using an Instron 3342 universal testing system (Instron Germany), with a stainless steel circular stamp (d = 4 mm) generating a force signal ranging from 8 N to 20 N with a frequency of 0.5 Hz along the z-axis. The force was applied with a speed of 2 mm/s for 5 cycles per measurement. The generated current, I, was converted into a voltage signal using a transimpedance amplifier (TIA) and recorded with a data acquisition system (SIRIUS Multi, Dewesoft). The charge *Q* was calculated from the calibrated V-I signal via integration. Figure 1b shows a schematic of the measurement set-up. To assess the reliability of the results, measurements were performed on multiple samples, originating from different MLD depositions. Furthermore, multiple measurements were performed on each sample for each employed force.

## 3. Results and Discussion

### 3.1. Growth of Zincone and Calcination to Porous ZnO

**Figure 2:**
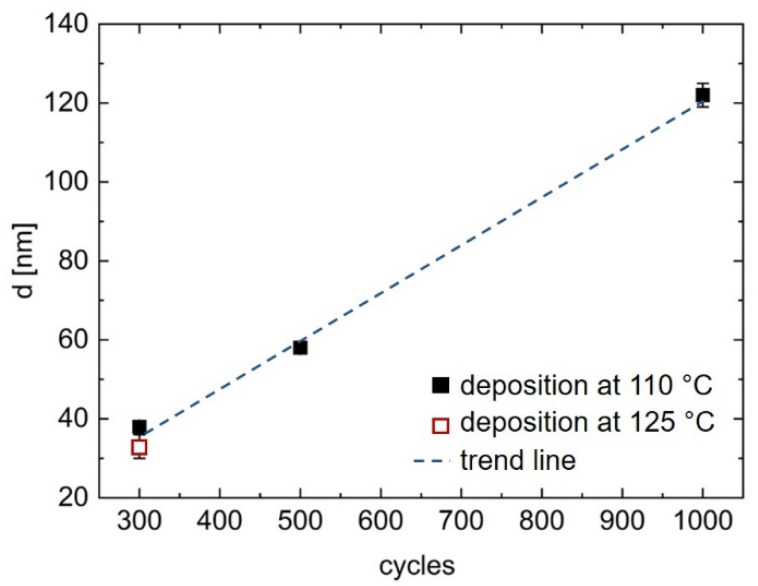
Thickness of zincone as a function of the number of deposition cycles measured with spectroscopic ellipsometry. The correlation value of the linear fit is r = 0.996.

The thickness of the zincone layers obtained by MLD was investigated ex situ via SE (Figure 2). To avoid the influence of ageing effects onto the thickness measurements, all samples were measured within 10 min of their first contact with ambient air.

The thickness of zincone deposited at 110 °C was determined for up to 1000 MLD cycles as a function of the number of cycles and exhibits a linear trend (Figure 2) with a correlation coefficient of r = 0.996. By simply dividing the overall thickness of the zincone layers through the number of employed MLD cycles, the growth per cycle (GPC) can be obtained, which was found to be (1.30 ± 0.05) Å/cycle for the samples deposited for 300 cycles. For higher cycle numbers, the GPC was found to be constant at 1.2 Å, with an increasing error up to ±0.07 Å. Thus, the GPC lies within the uncertainties of all measured values over all investigated cycle numbers. Additionally, zincone deposited at a higher substrate temperature of 125 °C for 300 cycles yielded a lower GPC of (1.1 ± 0.04) Å/cycle.

In literature, a wide range of growth rates is reported for zincone layers from EG and DEZ. Peng et al. [29] found a GPC of 0.57 Å/cycle at 120 °C and of 0.39 Å/cycle at 165 °C for layers on oxidized Si. However, these values were measured on aged samples, which had lost thickness after being subjected to air. Yoon et al. [28] reported a GPC of 0.7 Å/cycle at 130 °C for zincone on Si. Other GPC values between 4 Å/cycle and 0.25 Å/cycle for substrate temperatures ranging from 90 °C to 170 °C, respectively, were obtained from measuring the first 10 cycles of zincone deposited on ZrO_2_ nanoparticles. Perrotta et al. [36] reported a GPC of 1.05 Å/cycle at a substrate temperature of 110 °C for depositions up to 500 cycles.

Variances in the GPCs reported in literature point toward the influence of different experimental factors on this quantity, e.g., the reactor geometry, the working pressure, the precursor flow rate, the purging and pulsing times within each cycle, and the type of substrate. For this reason, the GPC reported here is deemed reliable. Furthermore, the linearity of the GPC confirms the stability and reproducibility of the process.

That the GPC was found to be lower for a higher deposition temperature also aligns with trends reported previously in literature: Zincone film growth is believed to occur by both the surface chemistry and the diffusion of DEZ into the zincone film. The diffused DEZ molecules would be available to react during subsequent EG exposure. However, at higher deposition temperatures either less DEZ diffuses into the film or the DEZ molecules desorb faster than at lower temperatures. Additionally, EG “double reactions” occur at higher deposition temperatures, during which EG reacts with two surface reactive surface species instead of one, thus effectively terminating the polymer chain and not generating new hydroxyl sites for zincone film growth, which in turn leads to a lower GPC [28].

All reported GPCs are significantly below the nominal molecule length, as the length of an EG molecule alone is ~5.3 Å [29]. This effect is also observed for other metalcones for which linear alipathic organic precursors are used [23,25,40] and can be explained by the tilting of the molecules with respect to the sample surface.

To prove the successful synthesis of zincone, ex situ FT-IR measurements were performed on the samples directly after deposition (Figure S3a). Furthermore, the stability of the zincone thin films was investigated by monitoring the development of one sample deposited at 110 °C in ambient air over several days, as detailed in Figure S3a,b in the Supplementary Materials. The examination of aging zincone reveals that the thickness decreases rapidly within the first few hours after subjecting zincone to ambient air and continues to decrease more gradually until it has reached a stable state after about three days (Figure S3b). The aging of zincone in ambient air has been previously investigated and explained by hydrolysis of the film with consequent thickness reduction [29]. This is visible also by FT-IR: the spectra of zincone change over time due to hydrolysis by water penetrating the film in ambient air (Figure S3a).

**Figure 3:**
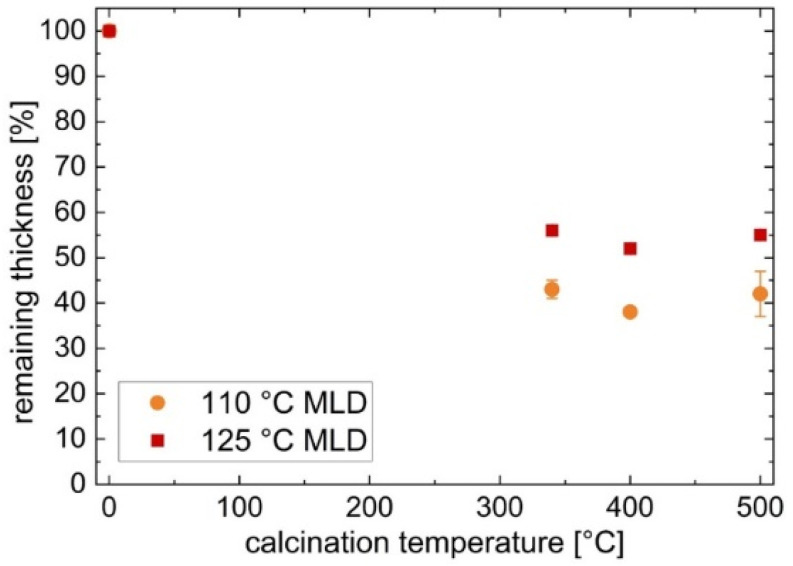
Thickness of molecular layer deposited zincone and percentage of the remaining thickness of ZnO after calcination from zincone at different temperatures. Zincone was obtained via molecular layer deposition (MLD) at either 110 °C or 125 °C.

**Figure 4:**
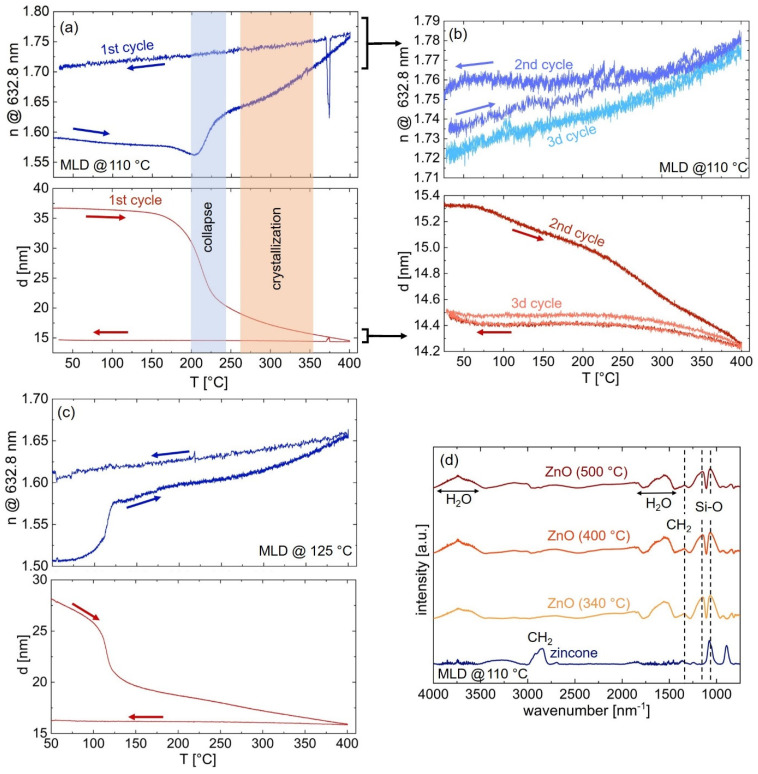
Calcination of zincone to obtain porous ZnO thin films. In situ spectroscopic ellipsometry follows the calcination of zincone deposited at (**a**,**b**) 110 °C (**c**) 125 °C. (**d**) Fourier transform infrared spectroscopy absorbance spectra of ZnO thin films calcinated at different temperatures and of the zincone prior calcination.

Calcination of the zincone films, which was performed to remove the organic content of the samples, was monitored by SE and FT-IR (Figure 3 and Figure 4).

The thickness values obtained from SE measurements after calcination reveal that 38–43% of film thickness remained for the samples deposited at 110 °C, whereas the 125 °C MLD deposition led to a remaining film thickness of 52–56% (Figure 3). Thus, the calcination of the samples results in a significant loss of thickness. Even though zincone deposited at a lower temperature yields a higher GPC, less of its original thickness is retained after calcination.

The development of the samples during calcination was investigated by in situ SE, in which the thickness and refractive index (@633 nm) were continuously tracked up to 400 °C (Figure 4a–c).

For the zincone deposited at 110 °C (Figure 4a), the thickness remains nearly constant until a steep decrease in thickness at about 200 °C, coinciding with a rapid increase in the refractive index. At higher temperatures, starting at about 225 °C, the thickness loss continues but becomes less steep, whereas the refractive index increases further. Another change in slope of the increasing refractive index can be spotted at about 300 °C, leading again to a more pronounced rise of the refractive index until the maximum temperature of 400 °C.

The first rapid thickness drop points to a collapse of the hybrid structure inside the sample, which can be explained by the removal of the organic content. Since the removal of hydrogen and carbon combined with the collapse of the film would cause an increased electron density, this would also explain the rapid increase in the refractive index at the same temperature. The slow thickness decrease at higher temperatures corresponds to further removal of hydrogen and carbon combined with reorientation processes of the remaining ZnO. The change in slope of the refractive index above 300 °C can be attributed to the onset of crystallization processes inside the sample (Figure 4a). In situ X-ray diffraction studies have previously reported Bragg peaks, which correspond to polycrystalline ZnO, at calcination temperatures of 340 °C [36].

As the sample is cooled, the thickness remains the same, whereas the refractive index gradually decreases. The decrease in the refractive index during the cooling phase is an effect known for stable crystalline ZnO films [47]. The remaining ZnO exhibits a higher mass density and consequently a higher electron density, which also results in a higher index of refraction [37]. Compared to the literature, similar behavior has been observed for ZnO obtained from MLD zincone via calcination [36,37] or also for alumina from calcinated alucone films [48].

To test whether all of the organic content was removed from the samples during the calcination process, two additional heating cycles up to 400 °C were employed for a sample obtained from 110 °C zincone and followed in situ with SE (Figure 4b). During the second calcination cycle, the thickness decreases further by about 0.8 nm, whereas the refractive index increases by about 0.02. This behavior indicates that some organic content remained after the first calcination process and is removed during the second heating cycle as well as structural rearrangements taking place inside the sample. The third calcination cycle shows no lasting change in refractive index or thickness, showing that no further organic content is removed from the sample. The temporary rise in refractive index and decline of the sample thickness are likely due to thermal expansion since both parameters return to their initial value after cooling. Previous studies have shown via FT-IR measurements, that calcinating zincone at 600 °C with a heating rate of 200 °C/h was sufficient to remove all organic content during one heating cycle [36].

The zincone sample deposited at 125 °C (Figure 4c) exhibits its first thickness collapse at about 120 °C and therefore at a significantly lower temperature than the zincone obtained from the MLD process at 110 °C. As the zincone samples are unstable in ambient air (Figure S3), a structural collapse at a calcination temperature that is lower than the temperature used during a deposition in a vacuum is possible. Again, this initial collapse is followed by a more gradual thickness decrease and a more moderate increase in the refractive index until the final calcination temperature of 400 °C. The change in slope of the increasing refractive index, which points toward crystallization processes inside the sample, occurs at about 300 °C, matching the behavior of the zincone deposited at a lower temperature. As the sample is cooled down, the thickness remains constant, whereas the refractive index decreases.

Yoon et al. [28] showed that changing the MLD process temperature leads to changes in the mechanism of the zincone deposition: at low temperatures (e.g., 90 °C), the EG mainly reacts with the available DEZ molecules on one side of the chain, thus creating new ZnOCH_2_CH_2_OH* hydroxyl sites available for subsequent reactions with DEZ. Additionally, a significant amount of DEZ diffuses into the already existing zincone layer, being accessible for reactions during subsequent exposure to EG to form new zincone polymer chains. These factors led to a relatively high GPC of 4 Å/cycle. In contrast, it was found that at high deposition temperatures (e.g., 170 °C) EG mainly undergoes “double” reactions, reacting with two ZnCH_2_CH_3_* species, consequently terminating the polymer chain. Additionally, at higher temperatures, less DEZ may diffuse into the zincone film and may also desorb at a faster rate from the film. This does lead to a lower GPC of 0.25 Å/cycle as well as differences in zincone composition. The changes in the composition of zincone depending on the MLD deposition temperature can not only explain that the main thickness collapse occurs at different calcination temperatures for zincone deposited at 110 °C (Figure 4a) and 125 °C (Figure 4b), but also that the remaining thickness of the calcinated ZnO differs up to 14% (Figure 3) with respect to the two employed MLD deposition temperatures.

In order to discern the influence of the calcination process on the chemical nature of the thin films, ZnO layers, which were obtained after one calcination cycle from zincone deposited at 110 °C, were investigated via FT-IR (Figure 4d). In comparison to zincone, the CH_2_ peaks at 2995–2785 cm^−1^ have vanished for the calcinated samples for all employed calcination temperatures. The broad peaks at 3500–3000 cm^−1^ and at 1780–1420 cm^−1^ can be assigned to water absorbed inside the ZnO thin films. The peak at 1070 cm^−1^ could be assigned to C–O stretching in C–C–O, but more probably it belongs to the Si–O–Si asymmetric stretching, together with 1160 cm^−1^ [37]. Si–O–Si is a mode that usually spreads over a wavenumber range of 950 cm^−1^ to 1200 cm^−1^ [49,50]. Unfortunately, the ZnO modes, which would be expected at around 400 cm^−1^ [36], cannot be detected as the instrument limit lies above. Around 1400–1300 cm^−1^ small modes remain, which can be assigned to CH_2_. This indicates that the removal of organic material is not complete after the first calcination process, which confirms the findings of the in situ SE measurements (Figure 4b).

### 3.2. Morphological, Chemical, and Crystallographic Characterization of Zinc Oxide

**Figure 5:**
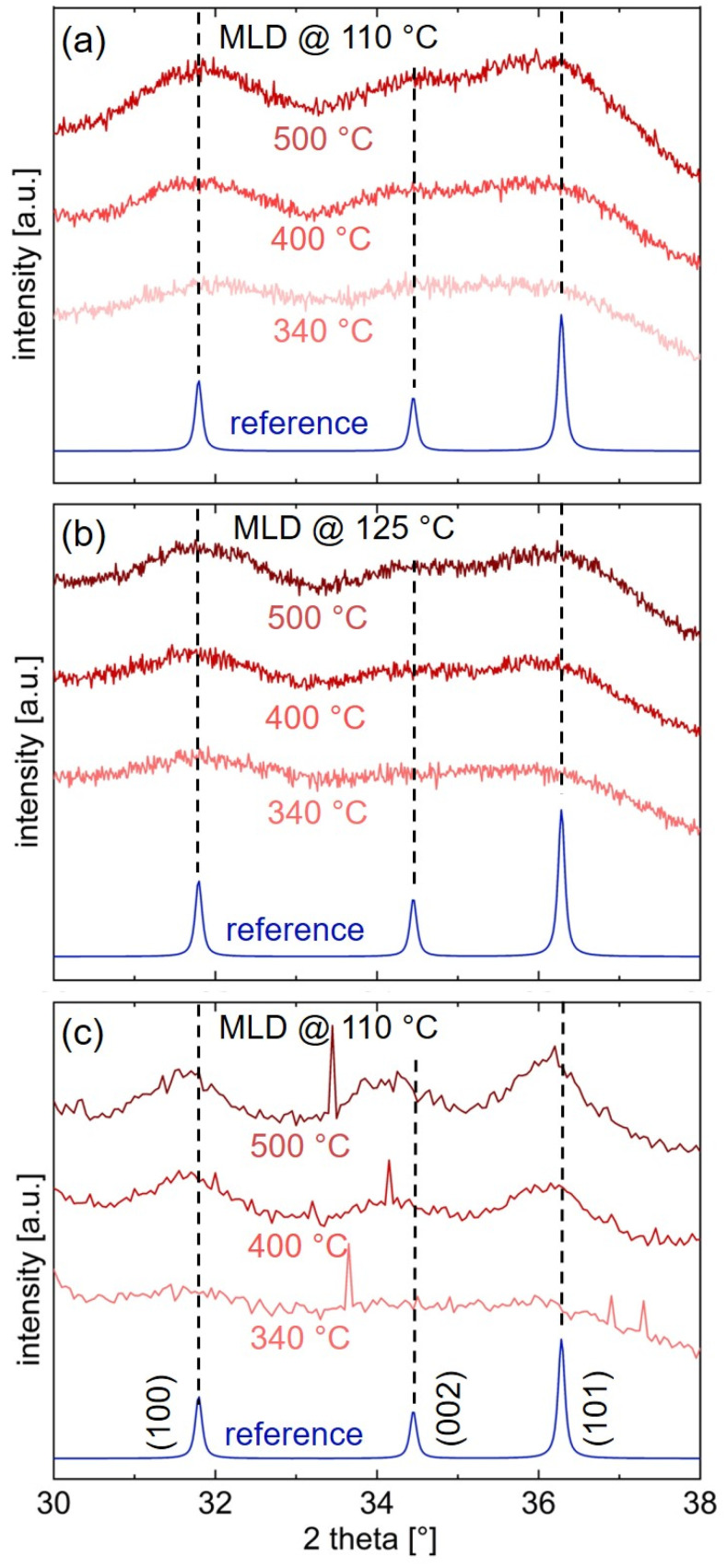
X-ray diffraction data of porous ZnO thin films on silicon. The ZnO was obtained from zincone (**a**) deposited at 110 °C, (**b**) deposited at 125 °C by molecular layer deposition, and subsequently calcinated at different temperatures. (**c**) Grazing incidence X-ray diffraction of ZnO obtained from calcinated zincone, which was deposited via molecular layer deposition at 110 °C. The narrow spikes are measurement artifacts resulting from instabilities of the detector. The reference corresponds to the calculated powder pattern of ZnO [51].

X-ray diffraction studies were performed on the calcinated layers (Figure 5). Figure 5a reports 2θ scans of ZnO derived from zincone deposited at 110 °C. Bragg peaks of crystalline ZnO appear at angles of 31.7°, 34.3°, and 36.3°, which correspond to the (100), (002), and (101) net planes of ZnO, respectively, when compared with the ZnO reference data [51]. With increasing calcination temperature, the peaks grow in intensity and in tandem decrease in width, suggesting an increase in crystallite size. ZnO obtained by calcinating zincone from a 125 °C MLD deposition also exhibits Bragg peaks for all calcination temperatures, which correspond to the (100), (002), and (101) orientation of ZnO and grow in intensity as the calcination temperature increases (Figure 5b). The in-plane orientation of ZnO samples obtained from 110 °C zincone was probed by GIXD and, as well, shows three Bragg peaks, referring to the (100), (002), and (101) planes (Figure 5c). Again, the GIXD peaks rise in intensity with increasing calcination temperature.

The observation of several Bragg peaks proves that ZnO has been obtained in its polycrystalline form via calcination of zincone. Additionally, the fact that the peak area (e.g., of the (100) peak) rises in tandem with the calcination temperature, reveals that the crystallization grows more prominent with increasing calcination temperature. This behavior is in agreement with previous studies of calcinated zincone-like layers [35,36].

From the full-width-half-maximum of the (100) and (101) Bragg peaks visible in the 2θ and GIXD measurements, the crystallite length of the ZnO thin films perpendicular and parallel to the sample surface was estimated with the Scherrer formula [52]. For the ZnO derived from 110 °C zincone after calcination to 500 °C, the out-of-plane crystallite size was determined to (8 ± 2) nm for the (100) peak and to (10 ± 2) nm for the (101) peak. The crystallite sizes parallel to the substrate lie between (11 ± 2) nm (for the (100)-plane) and (12 ± 2) nm (for the (101)-plane) for the same sample. Similar crystallite lengths of (7 ± 2) nm ((100)-plane) and (10 ± 2) nm ((101)-plane) were found for the ZnO sample obtained from 125 °C-zincone after calcination to 500 °C. Thus, the obtained ZnO crystallite sizes are comparable for both MLD deposition temperatures. Furthermore, the crystallites are slightly larger in-plane than out-of-plane. All calculated crystallite lengths lie within the thickness of the ZnO layers as determined via SE, namely (16 ± 2) nm and (18 ± 0.3) nm for ZnO derived from 110 °C zincone and from 125 °C zincone, respectively, after calcination to 500 °C.

No conclusions can be made about potentially remaining carbon in the calcinated samples from the XRD data presented in Figure 5, however, FT-IR measurements of the MLD-derived ZnO (Figure 4d) reports peaks corresponding to residual organic groups. Consequently, the carbon residuals do not inhibit crystal ripening processes during calcination.

**Figure 6:**
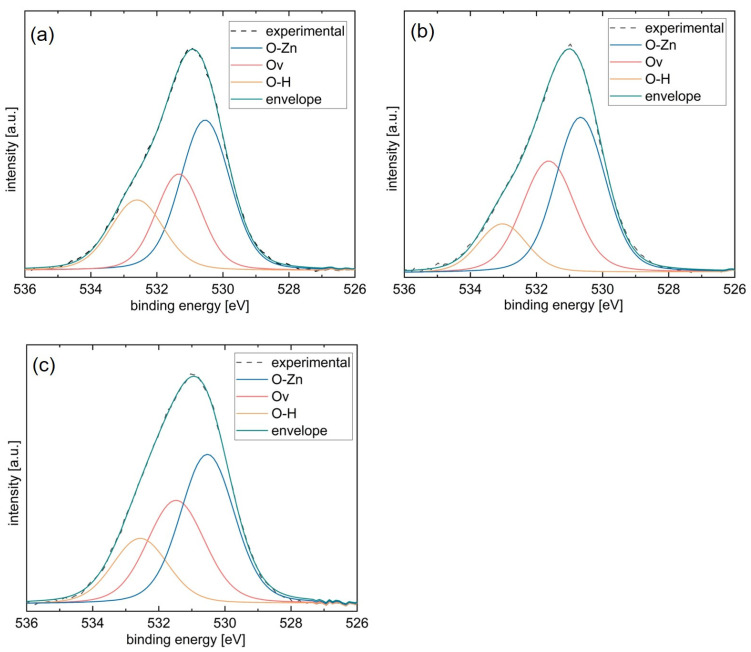
High-resolution O1s peak of ZnO thin films obtained from zincone deposited via molecular layer deposition at 110 °C and calcinated at (**a**) 340 °C, (**b**) 400 °C, (**c**) 500 °C. The experimental peak is decomposed into three Lorentz–Gaussian peaks, which correspond to different binding states of oxygen.

Information about the chemical composition of ZnO thin films synthesized from 110 °C zincone was obtained via XPS (Table 1, Figure 6).

In Table 1, the atomic content of carbon, oxygen, and zinc are reported in atomic percent, as obtained from XPS survey scans of the samples. For the pristine samples, a significant amount of carbon is detected, which is reduced to about 1% for all investigated samples after sputtering. This shows that a great portion of the carbon measured for the non-sputtered samples was a result of surface contamination. Actually, only a small percentage of carbon remains in the samples after calcination. This result agrees with the findings of FT-IR and in situ SE temperature studies presented in Figure 4 in the main file. The Zn/O ratio also changes markedly with the surface sputtering of the samples. After sputtering, the Zn/O ratio is found to be above unity. This is caused by preferential sputtering, which occurs when atomic masses of the measured elements are very different, as is the case for Zn and O. The influence of preferential sputtering can be corrected by normalizing the ratio to the values obtained from bulk crystalline ZnO in the order of 0.3 [53]. When applying the correction value, the Zn/O ratios of all measured samples lie within (1.0 ± 0.1)%.

The high-resolution XPS spectra of O 1s, which of ZnO obtained from 110 °C zincone after sputtering, serve to inform about the chemical environment of the oxygen in the film (Figure 6). The oxygen peaks are asymmetric, indicating a multicomponent oxygen species in the calcinated films. Therefore, the O 1s peak was decomposed into three contributions, assigned to the oxygen–zinc bonds in the oxide lattice (O–Zn), zinc hydroxides and/or oxygen vacancies (Zn–OH/Ov), and the hydroxyl binding state (O–H) at binding energies of 530.5 eV, 531.4 eV, and 532.6 eV, respectively. The observed peak position of the O 1s peak at 530.9 eV is higher than the expected value of 129.5 eV [54]. However, this observation has also been previously made for ZnO obtained via atomic layer deposition [55] and is likely due to the reduced screening of the O 1s electrons from their nucleus by the electron charge density in the region of oxygen vacancies, which raises the effective nuclear charge and in consequence also the observed binding energy [56].

The highest contribution for all investigated samples stems from the O–Zn component. The left shoulder of the O 1s peak, corresponding to the O–H groups, is reduced for calcination temperatures above 340 °C. Nevertheless, the overall composition of the O 1s peak remains the same for all employed calcination temperatures.

**Figure 7:**
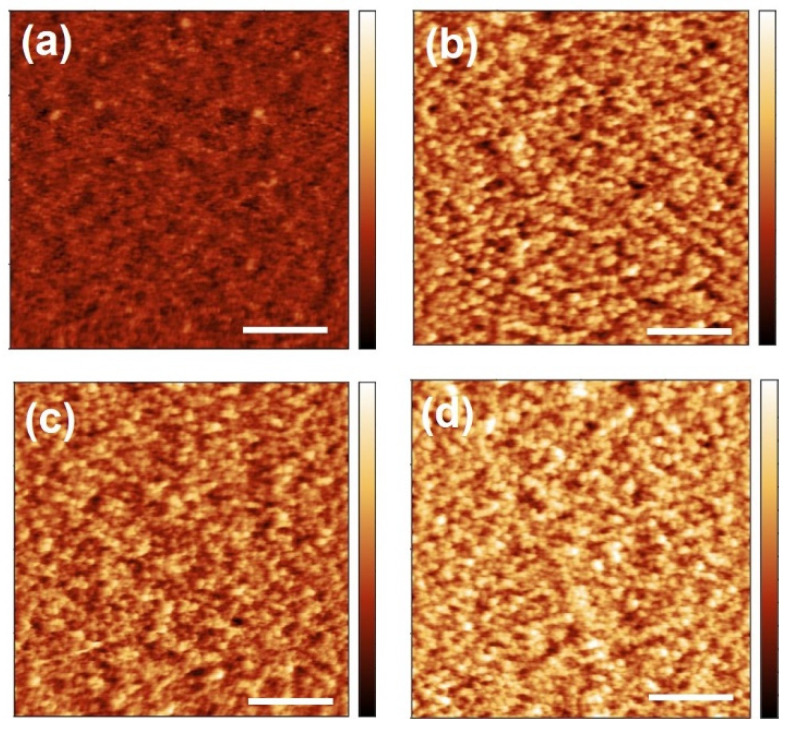
Atomic force microscopy topography images of porous ZnO thin films. The ZnO was obtained from zincone deposited by molecular layer deposition at (**a**) 110 °C and calcinated at 400 °C, as well as deposited at 125 °C and calcinated at (**b**) 340 °C, (**c**) 400 °C, (**d**) 500 °C. Each height scale spans from 0 to 7 nm; the white scale bar in each image corresponds to a length of 500 nm.

AFM images reveal that the surface of the ZnO thin films consists of regular structures (Figure 7), which are attributed to the ZnO crystallites. For the sample obtained from the MLD deposition at 110 °C (Figure 7a), the mean square roughness (σ_rms_) was determined to (0.5 ± 0.1) nm after calcination to 400 °C, and the average particle height to (2.0 ± 0.1) nm. The samples obtained from MLD at 125 °C exhibit higher σ_rms_-values of (1.0 ± 0.2) nm, (0.8 ± 0.2) nm, and (1.0 ± 0.2) nm after calcination to 340 °C, 400 °C, and 500 °C, respectively (Figure 7b–d). Therefore, the calcination temperature does not significantly influence surface roughness. In the same order, the average particle height of the samples obtained from MLD at 125 °C was determined to (3.5 ± 0.2) nm, (3.1 ± 0.1) nm, and (4.2 ± 0.2) nm. Although the average particle height is greater for the samples deposited at 125 °C, no clear effect of the calcination temperature on the average particle height can be discerned. The AFM images show large surface structures, whose lateral autocorrelation length obtained from Gaussian fitting of the height–height correlation function, is around 34 nm, for all samples.

### 3.3. Porosity Studies of Zinc Oxide

The open porosity of the ZnO was measured via ellipsometric porosimetry, utilizing water as the probing molecule. When presenting the adsorptive uptake via the Lorentz–Lorenz relationship as a function of the relative humidity, classical isotherms are obtained [36], which can be categorized according to the IUPAC classification: whereas a type I isotherm characterizes microporous materials, with pore diameters below 2 nm, a type IV isotherm represents mesoporous materials, with pore diameters between 2 and 50 nm [57]. Usually, real porous materials exhibit combinations of type I and type IV isotherms, since mesoporous materials also include a percentage of micropores [58].

**Figure 8:**
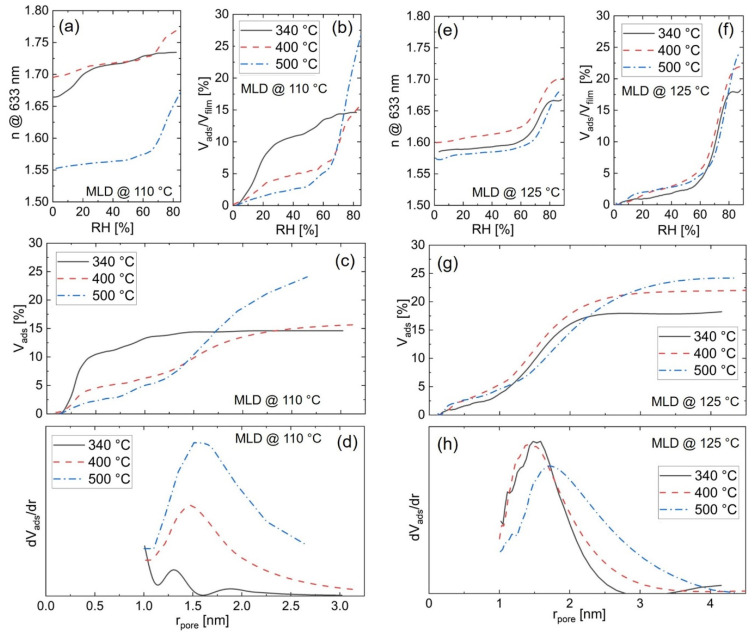
Ellipsometric porosimetry data of ZnO calcinated from zincone. ZnO was calcinated at different temperatures from 340 °C–500 °C; (**a**–**d**) depicts “110 °C-zincone” which was deposited at 110 °C and (**e**–**h**) “125 °C-zincone” deposited at 125 °C via molecular layer deposition. (**a**,**e**) Refractive index as a function of relative humidity; (**b**,**f**) adsorption isotherm; (**c**,**g**) volume fraction of pores filled with condensed adsorbate as a function of the pore radius; (**d**,**h**) pore size distribution for pore radii >1 nm.

The probed ZnO samples are derived from zincone deposited at 110 °C and 125 °C via MLD, which was subsequently calcinated at 340 °C, 400 °C, and 500 °C. The corresponding adsorption isotherms, volume fractions of pores filled with condensed adsorbate as a function of the pore radii, and pore size distributions for mesopores are reported in Figure 8.

For ZnO obtained from zincone deposited at 110 °C, the refractive index rises as the relative humidity is increased inside the measurement chamber for all investigated calcination temperatures (Figure 8a). However, the classical isotherms (Figure 8b) exhibit different shapes with respect to their calcination temperatures. For the isotherm of ZnO obtained after calcination at 340 °C, the steepest uptake of liquid inside the pores takes place between 5% and 20% relative humidity (RH). At higher RH the adsorption of the probing vapor is less pronounced. Since the adsorption at low humidity is solely attributed to nanopores and since the shape of the isotherm resembles a type I isotherm, the sample is thought to be mainly microporous. In contrast, for calcination temperatures of 400 °C and 500 °C, the main uptake of probing vapor inside the pores occurs between 60% and 80% RH. These curves match type IV isotherms, known for representing mesoporous materials. However, even for the higher calcination temperatures, some adsorption is detected at low RH values, indicating that a low percentage of micropores is also present in those samples.

Figure 8c depicts the adsorbed probe volume with respect to the pore radius, resulting in a cumulative function of the percentage of open pores of the investigated samples. For ZnO obtained from calcinating zincone at 340 °C, most of the adsorbate uptake occurs for pore radii smaller than 1 nm, reflecting that the majority of the pores lie in the microporous regime. In contrast, the samples calcinated at 400 °C and 500 °C exhibit the main part of their adsorption in the mesoporous regime.

**Figure 9:**
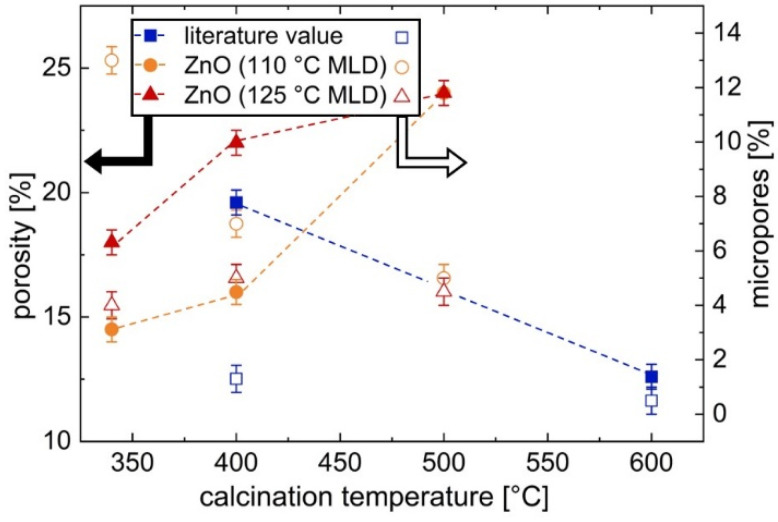
Total open porosity and amount of micropores of porous ZnO thin films. The ZnO was obtained from zincone, which was a molecular layer deposited at 110 °C or 125 °C, via calcination at different temperatures. The full symbols refer to the porosity, whereas the open symbols denote the micropore percentage; dashed lines serve as a guide to the eye. The symbols for the porosity for ZnO obtained at a calcination temperature of 500 °C overlap at 24% for both MLD temperatures. The results are compared to literature values [36].

With increasing calcination temperature, the microporosity steadily decreases, whereas the mesoporosity increases. In addition, the overall porosity increases with rising calcination temperature. The values for the total open porosity, as well as for the micropores are depicted in Figure 9.

The mean pore radius can be extracted from the pore size distribution, which is calculated via the derivative of the volume fraction of pores filled with condensed adsorbate with respect to the pore radius. The pore size distribution for ZnO derived from zincone deposited at 110 °C increases in width with increasing calcination temperatures (Figure 8d), with the mean pore radius in the mesoporous regime ranging from 1.3–1.5 nm, thus not changing significantly with calcination temperature. The maximum pore radius that can be measured with EP depends on the maximum relative humidity to which the samples are exposed. In this study, a max 90% RH was used, which means that only pores with radii below 11 nm pore can be measured. Nevertheless, previous studies from our group showed similar pore radii for zincone samples prepared with a similar procedure and exposed to RH up to 95% [36]. The porosity data were also confirmed by grazing incidence small angle X-ray scattering (GISAXS).

The amount of porosity that is introduced during the calcination results from a competition between the removal of organic content, which leads to a collapse of structure but also to the formation of voids and crystallization processes, which directly affect the arrangement and packing of the crystallites and thus the free volume in the ZnO films [36]. Therefore, the calcination temperature has a significant impact on the porosity of the samples.

Figure 8e–h shows the ellipsometric porosimetry results of ZnO obtained from zincone deposited at 125 °C. The overall porosity increases with increasing calcination temperature, as does the mesoporosity (Figure 8g and 9). Therefore, with regards to the calcination temperature, the porosity results of ZnO derived from 125 °C zincone follow the same trend as the porosity of ZnO calcinated from 110 °C zincone.

Figure 8f, which depicts the absorption isotherms of ZnO calcinated from 125 °C zincone, shows that the main surge of adsorbed adsorbate occurs between 60% and 80% for all employed calcination temperatures. Furthermore, all curves resemble type IV isotherms, suggesting that the samples contain mainly mesopores. Figure 8g, in which the volume fraction of pores filled with condensed adsorbate is reported, reveals that most of the probing vapor is indeed adsorbed in the mesoporous regime, for pore radii >1 nm.

The pore size distribution (Figure 8h) shows a mean pore radius ranging from 1.4–1.7 nm. The maximum total open porosity detected is 24% for the sample calcinated at 500 °C, with 19.5% mesopores. These values closely resemble the results from ZnO obtained from zincone deposited at 110 °C which was calcinated at the same temperature. However, for lower calcination temperatures, the porosity values deviate significantly with respect to the MLD process temperature. Zincone deposited at 125 °C and calcinated at 340 °C and 400 °C results in higher overall porosity and a lower percentage of micropores compared with their counterparts derived from 110 °C MLD (Figure 9).

Ergo, not only the calcination temperature can be used to influence the open porosity of the ZnO derived from zincone, but the MLD deposition temperature also plays an important role when it comes to the ZnO porosity. This behavior can be attributed to differences in the zincone compositions, which is a consequence of the different MLD process temperatures [28], as detailed in Section 3.2.

In the literature, porosity values up to about 20% were found for ZnO obtained from zincone deposited with EG and DEZ and subsequently calcinated at 400 °C. In contrast, a calcination temperature of 600 °C led to a lower porosity of about 13% [36]. Together with the data presented here, a more complete picture can be assembled of the influence of the calcination temperature on the porosity of the ZnO as depicted in Figure 7. It appears that the ideal calcination temperature lies around 500 °C, as this temperature leads to the highest overall porosity value (24% for both MLD process temperatures). Lower temperatures (e.g., 340 °C) result in less porosity due to remaining organic species and in a higher percentage of microporosity, as the micropores have not yet agglomerated to larger pores during rearrangement or crystallization processes. However, higher temperatures (e.g., 600 °C) also result in a lower porosity (Figure 9), presumably due to advanced crystallization processes.

### 3.4. Piezoelectric Characterization of Porous Zinc Oxide

To assess the macroscopic piezoelectric properties of the ZnO, three zincone samples deposited at 110 °C and calcinated at 400 °C were subjected to periodic force cycles with forces between 8 N to 20 N and the resulting piezoelectric current signal was measured as a function of time. A schematic of the measurement set-up is depicted in Figure 1b. XRD studies reveal a polycrystalline structure for the porous ZnO thin film on ITO/glass (Figure S4), similar to the one found for porous ZnO on silicon (Figure 5).

**Figure 10:**
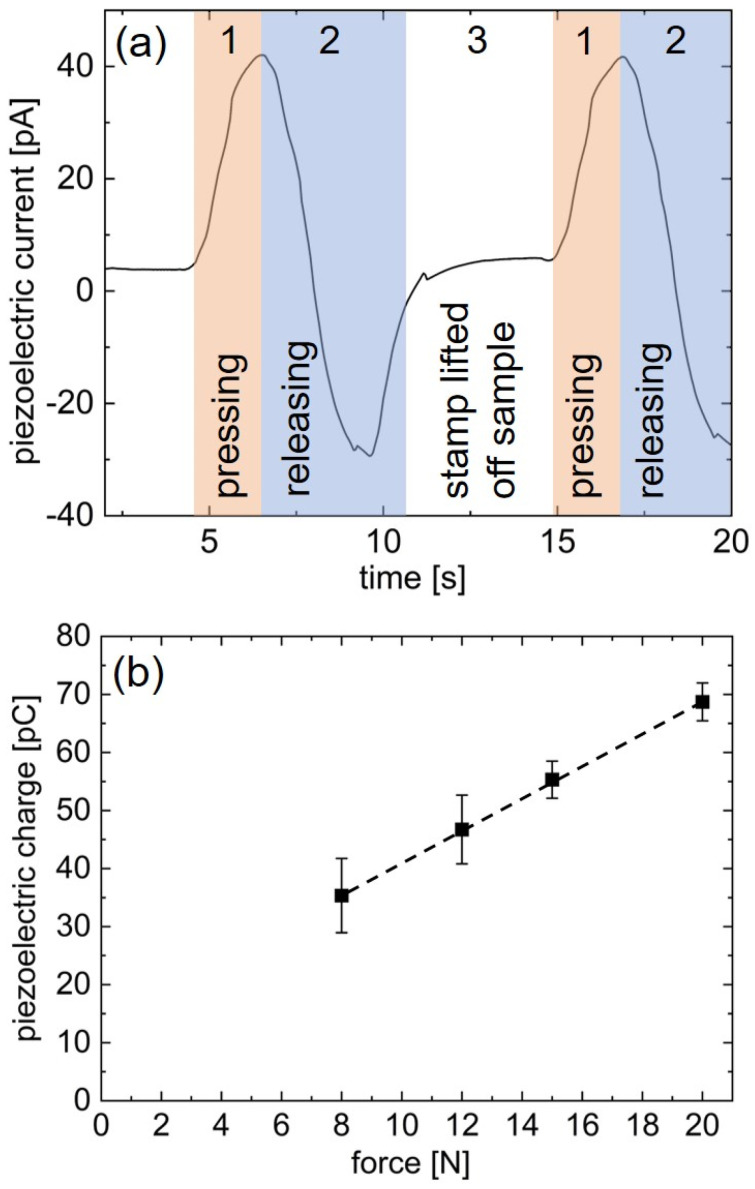
Piezoelectric measurements on porous ZnO thin films calcinated at 400 °C from zincone deposited via MLD at 110 °C. (**a**) Piezoelectric current signal versus time, measured for an applied force of 20 N; (**b**) generated piezoelectric charge as a function of the applied force.

Figure 10a presents a characteristic example of the recorded current response for one of the investigated ZnO samples subjected to five subsequent measurement cycles of 20 N. Each measurement cycle consists of three distinct stages: Positive current pulses are generated upon pressing onto the sample (stage 1) and negative current pulses upon releasing the force employing stamp from the sample (stage 2). At a certain point, the stamp is completely removed from the sample surface (stage 3), resulting in a relaxation of the measured current towards zero. However, this relaxation does not occur instantaneously but instead takes several seconds.

This behavior can be attributed to the elasticity of the system, which likely stems from the dielectric pEGDMA layer. Additionally, the small recoil current detected at the beginning of the relaxation phase is thought to be an artifact of the elasticity of the sample and has been previously observed, e.g., when depositing on flexible substrates [39]. The next measurement cycle begins as the stamp once more increases its force upon the sample surface and consequently the measured piezoelectric current rises again (stage 1).

The average piezoelectric peak current generated from one cycle depends on the employed force and ranges from (20 ± 2) pA for 8 N to (35 ± 2) pA for 20 N.

The piezoelectric charge, Q, can be calculated by integrating the piezoelectric current over time.

The obtained piezoelectric charge is depicted in Figure 10b as a function of the employed force. The piezoelectric charge increases in accordance with the employed force exhibiting a linear response, up to a charge of (69 ± 3) pC when subjected to 20 N. At lower forces, e.g., 0 N, an offset in the piezoelectric charge can be observed, which would not occur in an ideal system. This offset could be due to a slight overshoot in the applied force of the measurement system.

Since the ZnO films deposited on glass are not prone to bending, the piezoelectric coefficient can be calculated via

d_33_ = Q/F (1)

with Q as the piezoelectric charge and F as the excitation force.

From the slope of the linear fit in Figure 8b, the piezoelectric coefficient is determined to d_33_ = 2.8 pC/N. Although this piezoelectric coefficient is significantly lower than that of bulk wurtzite ZnO (d_33_ = 11.67 pC/N) [59], it is well comparable to previously reported values for thin films. ZnO thin films synthetized via plasma enhanced atomic layer deposition (PE-ALD) at 25 °C, which exhibited a polycrystalline structure similar to the ZnO thin films investigated here as well as a similar thickness, yielded a piezoelectric coefficient of 3.0 pC/N [39]. However, it should be noted that they obtained significantly higher piezoelectric coefficients for deposition onto flexible substrates and for higher deposition temperatures, which switched the preferred orientation of the PE-ALD ZnO films to (002). A different study, also concerned with nonporous ZnO thin films of a comparable thickness (50 nm), reported a significantly higher piezoelectric coefficient of d_33_ = (25 ± 15) pm/V for samples prepared on silicon wafer by pulsed laser deposition (PLD) [60]. In contrast to the present study, the piezoelectric response of the PLD samples was not assessed with a macroscopic stamp but instead microscopically via piezoresponsive force microscopy.

Table 2 shows a comparison on the piezoelectric properties of ZnO films and non-films with the ones of the present study.

No literature exists so far, regarding the assessment of the piezoelectric properties of porous ZnO thin films with a comparable thickness to the ones investigated in the present study. One recent study investigated porous ZnO films with a thickness of approximately 1 μm, prepared by radio frequency magnetron sputtering and subsequent calcination [11]. Not only the thickness but also the average pore size of those films differ significantly from the ones presented in this manuscript, ranging from about 70 nm to about 150 nm after calcination at 650 °C and 850 °C, respectively. Compared with their non-porous counterparts the study found a two-fold enhancement for the piezoelectric coefficient of the porous ZnO films, increasing from 1.12 pm/V to 2.62 pm/V. This behavior was ascribed to the lattice contraction in the c-direction induced by the pores.

One might expect that the porosity influences the piezoelectric response of ZnO compared with its non-porous equivalent due to the increased compressibility of the system and because of the air inside the pores, which could lower the electrical conductivity of the ZnO thin films. However, the open porosity decreased significantly to only 0.3% after deposition of the dielectric pEGDMA layer on the porous ZnO film (Figure S5), which was required to reduce current leakage during the measurements.

Nevertheless, the presented results showcase that MLD-derived ZnO has the potential to be employed as piezoelectric thin films in corresponding applications.

## 4. Conclusions

The chemical, morphological, crystallographic, and piezoelectric properties of porous ZnO thin films, derived from MLD zincone, were investigated.

Zincone thin films were deposited via a two-step MLD process, utilizing EG and DEZ as precursors, at 110 °C as well as 125 °C. The zincone layers were subsequently calcinated in the air at 340 °C, 400 °C, and 500 °C.

The resulting porous ZnO thin films exhibit a polycrystalline structure that grows more prominent with increasing calcination temperature, showing (100), (002), and (101) planes as measured by XRD and GIXD. AFM studies revealed that the calcination temperature does not significantly influence the RMS surface roughness of the resulting ZnO films and that the MLD deposition temperature only contributes slightly to the roughness, yielding differences <1 nm.

The impact of calcination temperature and MLD processing temperature on the open porosity of ZnO thin films was investigated via ellipsometric. The open porosity of ZnO derived from zincone deposited at 110 °C ranged from 14.5% to 24 %, rising with the calcination temperature from 340 °C to 500 °C. ZnO obtained from 125 °C MLD yielded a higher porosity of 18% after calcination at 340 °C. In addition, the mesoporosity was found to be over 12% greater for the ZnO obtained from 125 °C zincone than for the ZnO derived from 110 °C zincone after calcination at 340 °C. However, for the highest employed calcination temperature, the overall open porosity was found to be the same for both MLD temperatures, namely 24%. The pore size distribution showed an average between 1.3–1.7 nm for all measurements, depending slightly on the calcination temperature and the zincone deposition temperature.

When subjected to piezoelectric measurements, the ZnO thin films responded to the employed forces with a linearly increasing generated charge of up to (69 ± 3) pC when subjected to 20 N. Their piezoelectric coefficient, d_33_, was determined to 2.8 pC/N. These results may open new applications for the MLD-derived ZnO in the fields of piezoelectric sensing and energy harvesting.

## Figures and Tables

**Table 1:** Elemental compositions and Zn/O ratios of ZnO films prepared from zincone deposited by molecular layer deposition at 110 °C and calcinated at different temperatures. Values were determined for pristine and for surface sputtered samples from X-ray photoelectron spectroscopy survey scans. T_calc_ (°C) calcination temperature in degree Celsius.

T_calc._ (°C)	Sample Condition	Oxygen (at%) (±1%)	Zinc (at%) (±1%)	Carbon (at%) (±1%)	Zn/O Ratio (±0.1%)	Corrected Zn/O Ratio
340 °C	pristine	34	18	48	0.55	1.0 ± 0.1
	sputtered	43	56	1	1.31	
400 °C	pristine	37	27	36	0.73	1.1 ± 0.1
	sputtered	42	57	1	1.35	
500 °C	pristine	39	37	24	0.97	0.9 ± 0.1
	sputtered	45	54	1	1.21	

**Table 2:** Comparison of the d_33_ coefficient measured in this study with literature.

Thickness (nm)	Porosity	d_33_ (pC/V)	Ref.
60	yes	2.8	Present study
50	no	3	[39]
50	no	25 ± 15	[60]
1000	yes	2.62	[11]
1000	no	1.12	[11]
Nanowires	yes	2.89	[12]

## Data Availability

The data presented in this study are available on request from the corresponding author.

## Supplementary Materials

The following supporting information can be downloaded at: https://www.mdpi.com/article/10.3390/ma15196786/s1, Figure S1: Schematic of the MLD set-up and sections of the pressure recording of MLD depositions performed at 110 °C and 125 °C; Table S1: Overview of the processing temperatures of the molecular layer deposition set-up; Figure S2: Schematic of the measurement set-up for ellipsometric porosimetry; a detailed description about the custom initiated chemical vapor deposition set-up; Figure S3: Investigation of the stability of zincone via infrared spectroscopy and spectroscopic ellipsometry; Figure S4: X-ray diffraction data of one sample used for piezoelectric tests: 2θ-scan of ZnO on ITO/glass and covered with EGDMA; Figure S5: Ellipsometric porosimetry data of porous ZnO coated with ethylene glycol dimethacrylate. References [26,29] are cited in the supplementary materials.
