# Supplementary material for: Tuning the Porosity of Piezoelectric Zinc Oxide Thin Films Obtained from Molecular Layer-Deposited “Zincones”
Source: Materials (Basel). 2022 Sep 30;15(19):6786. doi: 10.3390/ma15196786 (PMC9572196; doi:10.3390/ma15196786)
Supplement: Supplementary file 1 [file materials-15-06786-s001.zip › materials-1877882-supplementary.pdf]

SUPPLEMENTARY INFORMATION

Tuning the Porosity of Piezoelectric Zinc Oxide Thin Films obtained from Molecular Layer Deposited "Zincones"

Marianne Kräuter[a], Taher Abu Ali[a,b], Barbara Stadlober[b], Roland Resel[a], Katrin Unger[a], Anna Maria Coclite[a*]

* e-mail: anna.coclite@tugraz.at
[a] Institute of Solid State Physics, NAWI Graz, Graz University of Technology, Petersgasse 16, 8010 Graz, Austria
[b] MATERIALS-Institute for Surface Technologies and Photonics Joanneum Research Forschungsgesellschaft mbH, Franz-Pichler-Str. 30, 8160 Weiz, Austria

----

Ad Experimental:

Figure S1 and Table S1 contain details about the molecular layer deposition (MLD) process, additional to the information reported in the experimental section of the main file. Table 1S gives an overview of the employed processing temperatures in the MLD set-up. Figure S1 shows a schematic of the MLD set-up as well as snippets of the precursor pressure spikes recorded during the MLD process.
Figure S2 is concerned with the measurement set-up employed for ellipsometric porosimetry measurements. Details about the procedure are discussed in the main file in the experimental section.

Table S1. Overview of the processing temperatures of the molecular layer deposition set-up. A schematic of the set-up is depicted in Figure S1a.
T [°C] … temperature in degree Celsius

part of set-up	T [°C]
EG vessel	(76 ± 2) °C
EG line	(99 ± 2) °C
DEZ vessel	room temperature
DEZ line	(63 ± 1) °C
exhaust line	(95 ± 5) °C
reaction chamber – sample stage	(104 ± 4) °C - (110 ± 2) °C
	(119 ± 4) °C - (125 ± 2) °C

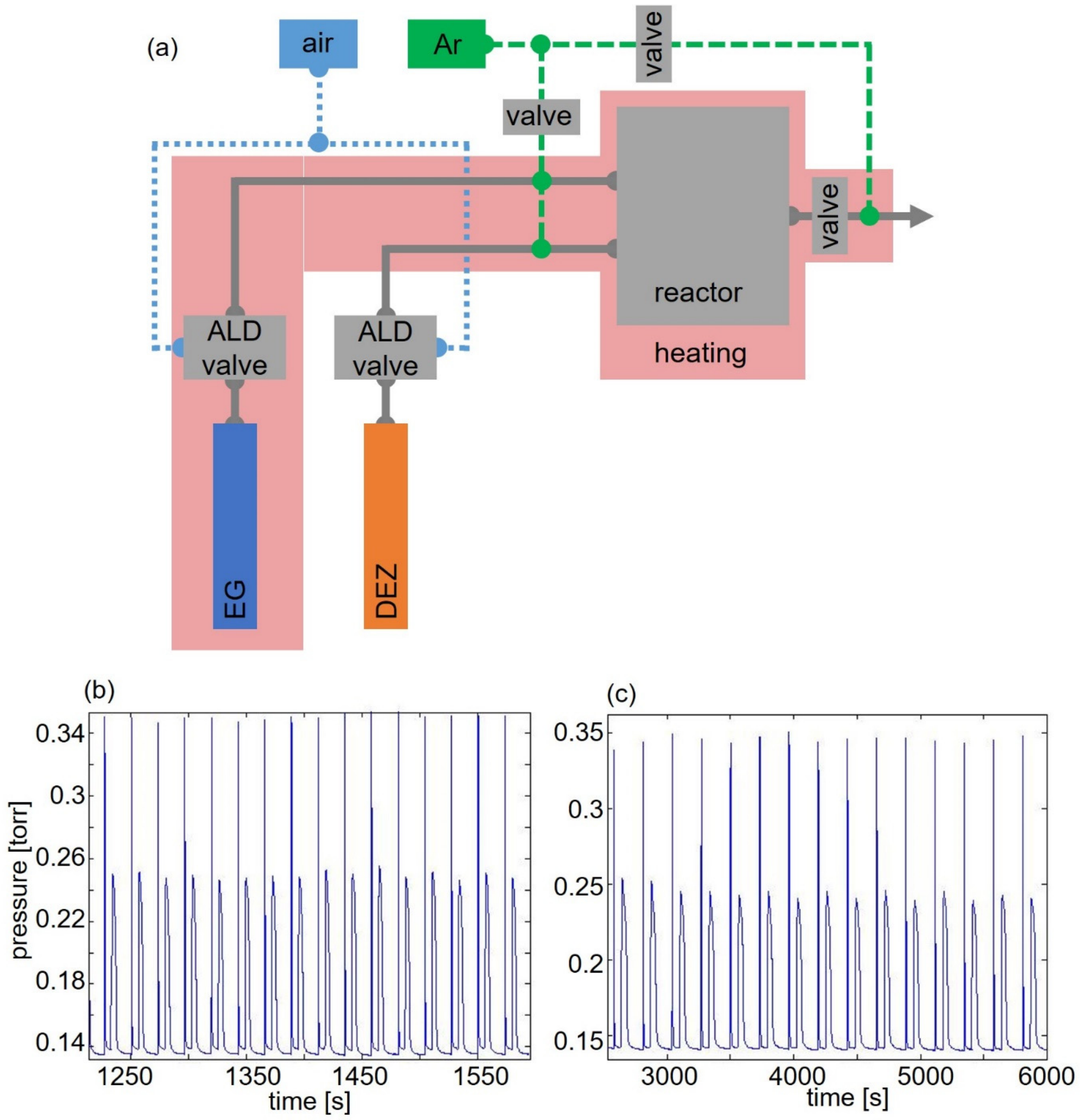

Figure S1. (a) Schematic of the set-up for molecular layer deposition; and a section of the pressure recording of a deposition performed at (b) 110 °C, (c) at 125 °C. The smaller pressure spikes (Δp = (0.1 ± 0.02) torr) correspond to EG, the larger pressure spikes (Δp = (0.2 ± 0.02) torr) to DEZ.

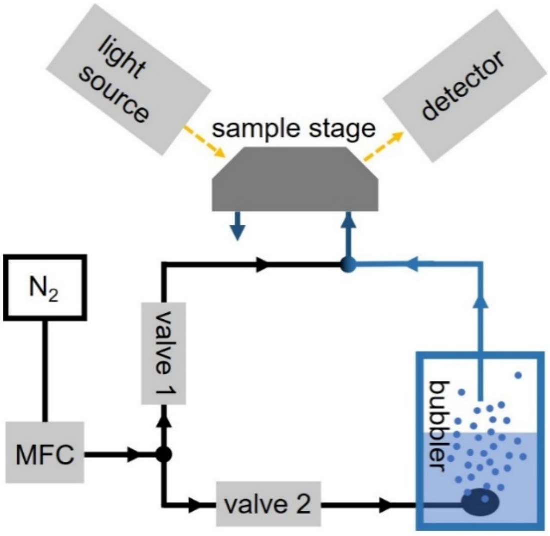

Figure S2. Schematic of the measurement set-up for ellipsometric porosimetry. “MFC” stands for “mass flow controller”. “valve 1” and “valve 2” correspond to manual needle valves, which can be opened or closed gradually.

The following description should provide more details about the custom iCVD set-up, that was employed to deposit pEGDMA layers onto porous ZnO films during the preparation of the samples for piezoelectric measurements:
The custom iCVD set-up consists of a cylindrical vacuum chamber with a glass cover, which was evacuated by a rotary vane pump (Pfeiffer Vacuum DUO65). The substrate temperature was controlled with aid of a chiller (Thermo Scientific Accel 500LC) connected to the bottom of the reactor. Glass jars, which contained the chemicals, were connected to the vacuum chamber via manual needle valves. The line system between the chemical jar of EGDMA and reactor was heated to about 100 °C to avoid condensation. The initiator was fed into the reactor chamber through a separate line. A nickel-chromium wire array (Goodfellow) inside the reactor near the top of the chamber can be heated by driving a current through it via a power supply (Heinzinger PTN 350-5) in order to break the initiator molecules apart and to start the polymerization process. The thickness of the growing polymer was controlled in-situ by monitoring the intensity change of a He-Ne laser beam (633 nm, ThorLabs), which was directed through the glass cover of the vacuum chamber onto a piece of Si wafer (Siegert Wafer) and reflected back into a detector which measured the intensity over time.

Ad Results:

The ageing of the molecular layer deposited zincone thin films in ambient air was investigated with FT-IR and spectroscopic ellipsometry.
The FT-IR spectrum in Figure S3a shows the typical absorption modes generally assigned to EG-DEZ zincones [26, 29]. Weak O-H stretching bands appear between 3500 and 3000 cm^{-1} showing the onset of hydrolyzation due to air exposure. The adsorption modes between 2995 cm^{-1} and 2785 cm^{-1} correspond to asymmetric (2920 cm^{-1}) and symmetric (2845 cm^{-1}) stretches of C-H in the CH_2-unit as well as combination modes (2690 cm^{-1}). The peak at 1365 cm^{-1} corresponds to the CH_2 wag mode, the one at 1250 cm^{-1} to the CH_2 twist. 1180 cm^{-1} is relatable to the C-C stretch mode and 1085 cm^{-1} is assigned to Si-O. At 895 cm^{-1} the Zn-O stretch absorbs. The areas between 3950 cm^{-1} and 3080 cm^{-1} as well as between 1780 cm^{-1} and 1420 cm^{-1} show H_2O-peaks, covering e.g. the CH_2 scissors mode expected at 1456 cm^{-1}.

Additionally, Figure S3a shows how the FT-IR-spectra of zincone change due to hydrolysis by water penetrating the film in ambient air. A rise in water- (1950-1420 cm^{-1}) and O-H-peaks (3500-3000 cm^{-1}) is detected. Additionally, a new peak appears at about 1140 cm^{-1} corresponding to C-O. It is noteworthy, that the Zn-O peak at 895 cm^{-1} diminishes with ageing.

The degradation of zincone also manifests in a loss of thickness, as observed via spectroscopic ellipsometry (Figure S3b). In the first few hours after subjecting the zincone to ambient air, the thickness decreases rapidly from 38 nm to 30.5 nm and continues to decrease more gradually until it has reached a stable state after about three days at about 29 nm.

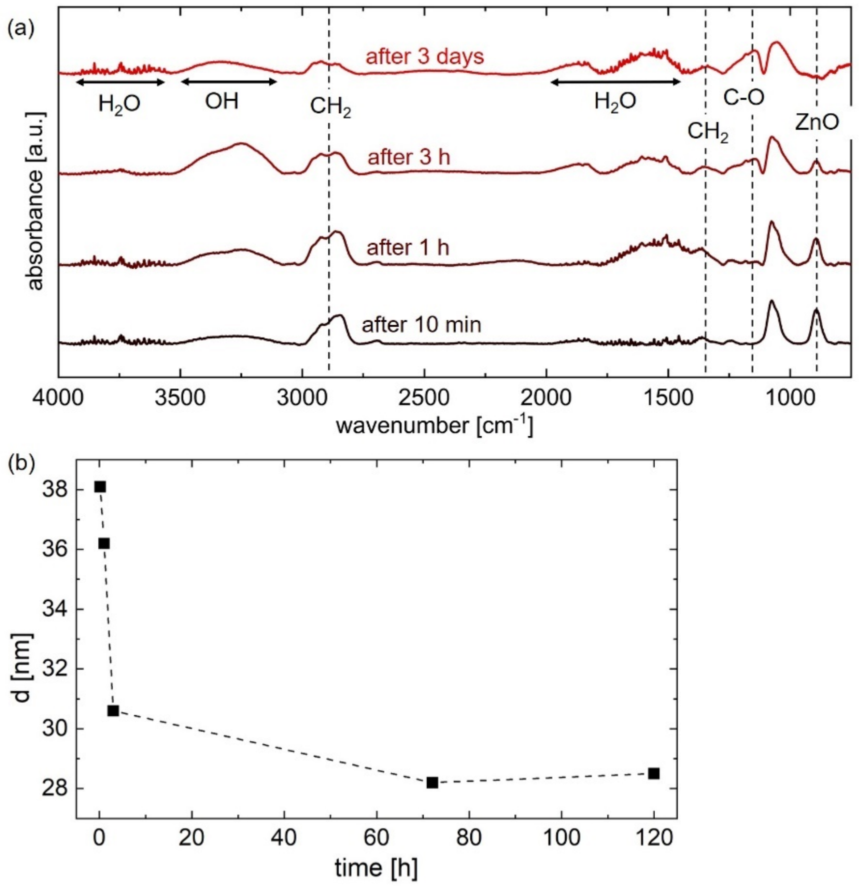

Figure S3. Investigation of the stability of zincone deposited via molecular layer deposition. (a) Fourier transform infrared spectroscopy measurements of zincone in ambient air for different time periods. (b) thickness of zincone in ambient air for different time periods, measured via spectroscopic ellipsometry.

Adding to the XRD studies presented in the main file (Figure 5), which studied the crystallinity of ZnO on silicon, Figure S4 shows a 2θ-scan of ZnO on ITO/glass and covered with approximately 60 nm of EGDMA. The ZnO was obtained via calcination at 400 °C from zincone, which had been molecular layer deposited at 110 °C. After adding Cu tape as a top electrode, the measured sample was employed for the piezoelectric measurements presented in the main file in Figure 10.

The thin film on ITO/glass exhibits diffraction peaks associated with the polycrystalline structure of ZnO. The left-shift of the diffraction peaks is presumably caused by stress, e.g. induced by the substrate.

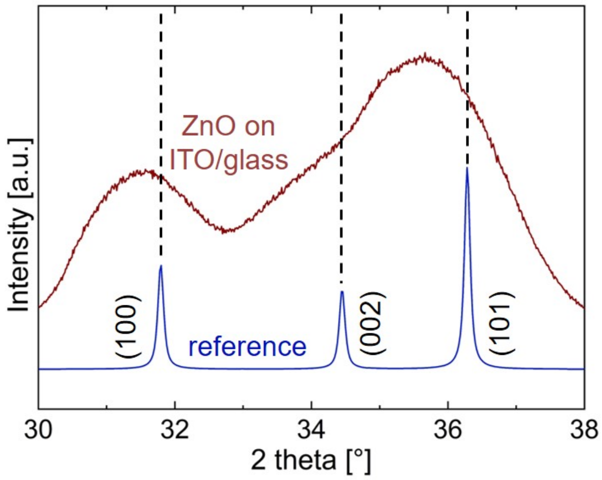

Figure S4. X-ray diffraction data of porous ZnO thin films on a glass substrate covered with ITO. Additionally, the ZnO is covered by approximately 60 nm Ethylene glycol dimethacrylate. The ZnO was obtained from zincone deposited at 110 °C by molecular layer deposition and subsequently calcinated at 400 °C. The reference corresponds to the calculated powder pattern of ZnO [52].

Figure S5 shows that the open porosity decreased of the ZnO layers decreased significantly to only 0.3% after deposition of the dielectric pEGDMA layer on top of them, which was required to reduce current leakage during the piezoelectric measurements.

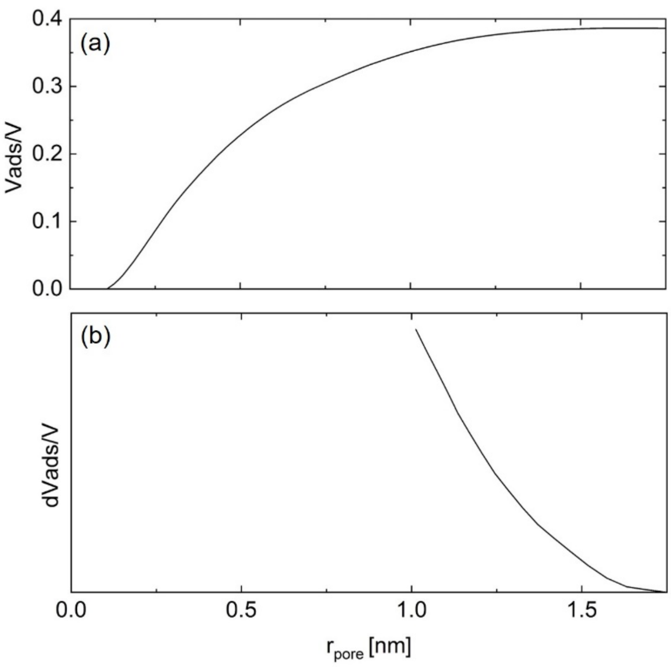

Figure S5. Ellipsometric porosimetry data of porous ZnO coated with ethylene glycol dimethacrylate. The ZnO was obtained via calcination at 400 °C from zincone, which was molecular layer deposited at 110 °C. (a) volume fraction of pores filled with condensed adsorbate as a function of the pore radius, (b) pore size distribution for pore radii > 1 nm.

Bibliography for Supplementary Information:

[28] Yoon, B.; O'Patchen, J.L.; Seghete, D.; Cavanagh, A.S.; George, S.M. Molecular layer deposition of hybrid organic-inorganic polymer films using diethylzinc and ethylene glycol. Chem. Vap. Depos. 2009, 15, 112–121, doi:10.1002/cvde.200806756.
[29] Peng, Q.; Gong, B.; VanGundy, R.M.; Parsons, G.N. "Zincone" zinc oxide - Organic hybrid polymer thin films formed by molecular layer deposition. Chem. Mater. 2009, 21, 820–830, doi:10.1021/cm8020403.
